# (2,2′-Bipyrid­yl)(η^6^-*p*-cymene)iodidoruthenium(II) hexa­fluorido­phosphate

**DOI:** 10.1107/S2414314623003929

**Published:** 2023-05-12

**Authors:** Monsuru T. Kelani, Alfred Muller, Koop Lammertsma

**Affiliations:** a Department of Chemical Sciences, University of Johannesburg, Auckland Park, Johannesburg 2006, South Africa; b Department of Chemistry and Pharmaceutical Sciences, Faculty of Sciences, Vrije Universiteit Amsterdam, De Boelelaan 1083, 1081 HV Amsterdam, The Netherlands; Purdue University, USA

**Keywords:** Ruthenium, *p*-cymene, 2,2′-bipyrid­yl, crystal structure

## Abstract

The title compound, having the mol­ecular formula [RuI(η^6^-C_10_H_14_)(C_10_H_8_N_2_)]PF_6_, crystallizes in the triclinic *P*  (*Z* = 2) space group as a half-sandwich complex resembling a three-legged piano stool. Important geometrical parameters include Ru—cymene centroid = 1.6902 (17) Å, Ru—I = 2.6958 (5) Å, [Ru—N]_avg_ = 2.072 (3) Å, N1—Ru—N2 = 76.86 (12)° and a dihedral angle between the planes of the two rings of the bipyridyl system of 5.9 (2)°. The PF_6_^−^ ion was treated with a twofold disorder model, refining to a 65.0 (8):35.0 (8) occupancy ratio. The crystal packing features C—H⋯F/I inter­actions.

## Abstract 2

The title compound crystallizes in the triclinic *P* 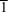 (*Z* = 2) space group as a half-sandwich complex resembling a three-legged piano stool. The crystal packing features C—H⋯F/I inter­actions.

## Graphical abstract

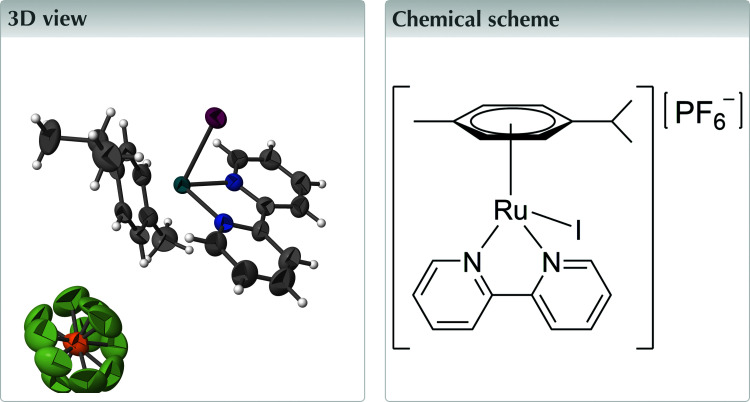

## Structure description

η^6^-Arene–ruthenium(II) complexes have demonstrated a high tendency to exhibit anti-tumour activity through DNA binding inter­actions (Colina-Vegas *et al.*, 2015 ▸; Yarahmadi *et al.*, 2023 ▸) and protein kinase inhibition (Atilla-Gokcumen *et al.*, 2006 ▸). In addition, they also exhibit catalytic properties, especially in the hydrogenation of ketones (Ngo & Do, 2020 ▸). The investigation of their structural properties will provide insight into the strategic design and development of new similar ruthenium half-sandwich complexes.

**Figure 1:**
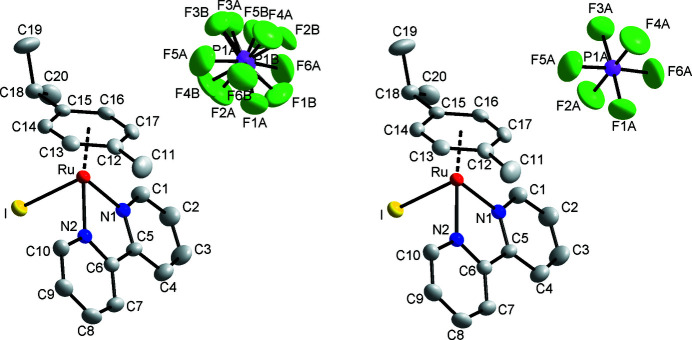
The molecular entities of the title compound with 50% probability displacement ellipsoids with and without the second component of the PF_6_^−^ disorder (hydrogen atoms are omitted for clarity).

**Figure 2:**
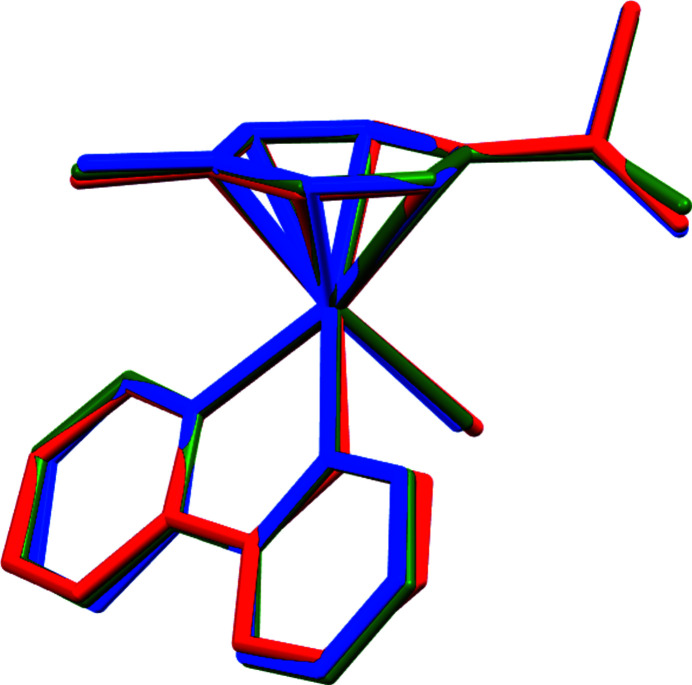
An overlay displaying the geometrical alignment between the title compound (in red) with the non-solvated chlorido analogue (Colina-Vegas *et al.*, 2015 ▸, in blue), and the methanol-solvated chlorido analogue (Wu *et al.*, 2008 ▸, in green) with r.m.s.d.s of 0.215 and 0.175 Å, respectively.

The title compound (Fig. 1 ▸) shows the typical piano-stool conformation with the *p*-cymene unit displaced by 1.6902 (17) Å from the central Ru^II^ atom, and the bipyridyl and iodido ligands taking up the remainder of the coordination sphere. The bond lengths of Ru—N1 [2.073 (3) Å] and Ru—N2 [2.072 (3) Å] are identical within experimental error, but were found to be slightly shorter than normal (CSD V5.43 September 2022 update, 785 entries with *p*-cymene-Ru—*N*,*N*′ bidentate; Groom *et al.*, 2016 ▸) in 1501 samples with a mean value of 2.11 (4) Å. The coordination environment is distorted from the ideal octa­hedral shape, primarily due to the pincer movement and twisting of the bidentate ligand [N1—Ru—N2 = 76.86 (12)°, dihedral angle between the two pyridyl moieties of the bipyridyl ligand = 5.9 (2)°]. The isopropyl group is eclipsed with the iodido group, similar to what is observed for the chlorido counterpart, reported as a non-solvated (Colina-Vegas *et al.*, 2015 ▸) and a methanol solvated form (Wu *et al.*, 2008 ▸), although the three crystal structures are not isostructural. A superimposed drawing of the iodido and chlorido complexes shows marginal deviations with the 2,2-bypiridyl and methyl group of the cymene ligand, resulting in an overall r.s.m.d. of 0.215 and 0.175 Å for the non-solvated (Colina-Vegas *et al.*, 2015 ▸) and methanol-solvated chlorido analogues (Wu *et al.*, 2008 ▸), respectively (see Fig. 2 ▸). The overlay is based on all non-hydrogen atoms except for the halogen atoms.

**Figure 3:**
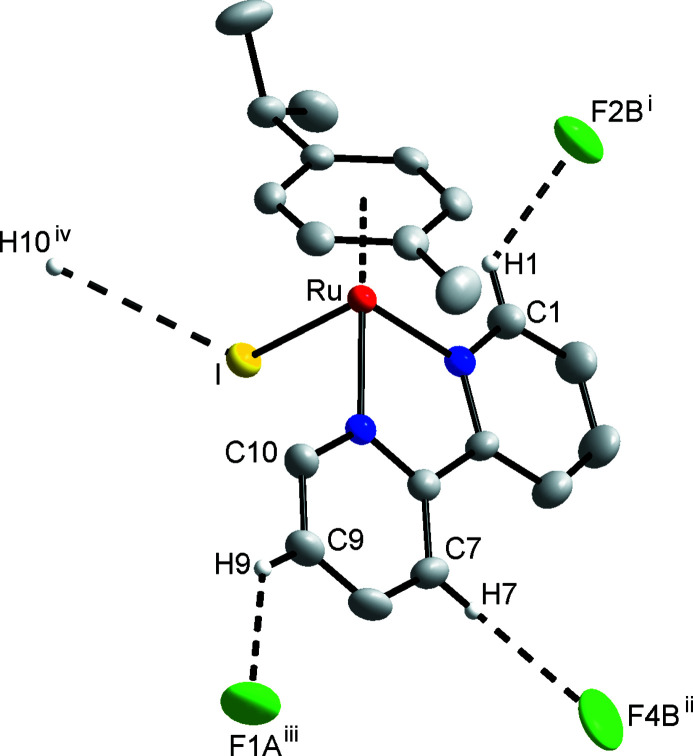
Non-classical hydrogen-bonding inter­actions observed in the crystal-packing arrangement between C—H and F, I. Symmetry codes: (i) −*x* + 2, −*y* + 1, −*z* + 1; (ii) *x* − 1, *y*, *z*; (iii) −*x* + 1, −*y*, −*z* + 1; (iv) −*x* + 1, −*y*, −*z* + 2.

Several non-classical hydrogen bonds exist between a C—H group (from the Ru complex) and the F atom of the PF_6_ anion, as well as one to an I atom of a neighbouring mol­ecule (Fig. 3 ▸ and Table 1 ▸). No discernible packing motifs were observed.

## Synthesis and crystallization

To a solution of (*p*-cymene)di­iodido ruthenium(II) dimer (200 mg, 0.20 mmol, 1 eq.) in methanol was added bi­pyridine (127 mg, 0.82 mmol, 4 eq.), resulting in the formation of an orange precipitate within 2 min. The reaction mixture was refluxed for 6 h, after which it was cooled to room temperature. NH_4_PF_6_ (100 mg, 0.61 mmol, 3 eq) was added and stirred for 1 h, and then concentrated *in vacuo*. The residue was purified by column chromatography using silica gel and the solvent system, CH_2_Cl_2_: MeOH = 99:1 (*R*_f_ = 0.36), as eluent to obtain an orange compound (108 mg, 0.20 mmol). The compound was crystallized by slow evaporation from a mixture of toluene and acetone. Yield, 99%, ^1^H NMR (500 MHz, DMSO-*d*_6_): δ 9.47 (*d*, *J* = 5.5 Hz, 2H), 8.65 (*d*, *J* = 8.0 Hz, 2H), 8.25 (*t*, *J* = 7.5 and 8.0 Hz, 2H), 7.75 (*t*, *J* = 6.0 and 7.0 Hz, 2H), 6.15 (*d*, *J* = 6.5 Hz, 2H), 6.01 (*d*, *J* = 6.0 Hz, 2H), 2.71 (*m*, *J* = 7.0 Hz, 1H), 2.40 (*s*, 3H), 0.97 (*d*, *J* = 7.0 Hz, 6H); ^13^C NMR (125 MHz, DMSO-*d*_6_): δ 155.70 (CH), 154.29 (C), 139.82 (CH), 127.46 (CH), 123.69 (CH), 103.65 (C), 103.57 (C), 86.60 (CH), 83.84 (CH), 30.30 (CH), 21.54 (CH_3_), 18.20 (CH_3_); ^13^C DEPT NMR (125 MHz, DMSO-*d*_6_): 155.47 (CH), 139.59 (CH), 127.23 (CH), 123.45 (CH), 86.37 (CH), 83.60 (CH), 30.06 (CH), 21.31 (CH_3_), 17.96 (CH_3_); FTIR (neat, cm^−1^): 2924, 2854, 1604 (C=C), 1442, 1381, 830, 763, 555.

## Refinement

Crystal data, data collection and structure refinement details are summarized in Table 2 ▸. The PF_6_ counter-ion had elongated thermal displacement ellipsoids and was treated using a twofold disorder model. Refinement of the disorder was kept stable with SADI distance restraints and ellipsoid sizes by SIMU with e.s.d.’s of 0.02 Å and 0.02 Å^2^, respectively. The distribution of the disorder model over the two sites was coupled to a free variable that will refine to unity for the two components. The final ratio was 65.0 (8):35.0 (8) for parts *A*:*B*.

## Supplementary Material

Crystal structure: contains datablock(s) global, I. DOI: 10.1107/S2414314623003929/zl4053sup1.cif

Structure factors: contains datablock(s) I. DOI: 10.1107/S2414314623003929/zl4053Isup3.hkl

CCDC reference: 2260167

Additional supporting information:  crystallographic information; 3D view; checkCIF report

## Figures and Tables

**Table 1:** Hydrogen-bond geometry (Å, °)

*D*—H⋯*A*	*D*—H	H⋯*A*	*D*⋯*A*	*D*—H⋯*A*
C1—H1⋯F2*B* ^i^	0.93	2.44	3.136 (13)	132
C7—H7⋯F4*B* ^ii^	0.93	2.65	3.41 (2)	140
C9—H9⋯F1*A* ^iii^	0.93	2.45	3.159 (7)	134
C10—H10⋯I^iv^	0.93	3.23	4.131 (5)	164

Symmetry codes: (i) 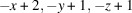 ; (ii) 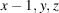 ; (iii) 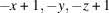 ; (iv) 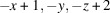 .

**Table 2:** Experimental details

Crystal data	
Chemical formula	[RuI(C_10_H_14_)(C_10_H_8_N_2_)]PF_6_
*M*_r_	663.33
Crystal system, space group	Triclinic, *P* 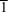
Temperature (K)	293
*a*, *b*, *c* (Å)	9.3020 (8), 10.4068 (9), 12.0732 (11)
α, β, γ (°)	86.046 (2), 82.838 (2), 88.953 (2)
*V* (Å^3^)	1156.80 (18)
*Z*	2
Radiation type	Mo *K*α
μ (mm^−1^)	2.14
Crystal size (mm)	0.39 × 0.24 × 0.08

Data collection	
Diffractometer	Bruker *APEX* DUO
Absorption correction	Multi-scan (*SADABS*; Krause *et al.*, 2015 ▸)
*T*_min_, *T*_max_	0.657, 0.746
No. of measured, independent and observed [*I* > 2σ(*I*)] reflections	41246, 4700, 3595
*R*_int_	0.060
(sin θ/λ)_max_ (Å^−1^)	0.625

Refinement	
*R*[*F* ^2^ > 2σ(*F* ^2^)], *wR*(*F* ^2^), *S*	0.032, 0.077, 1.05
No. of reflections	4700
No. of parameters	345
No. of restraints	312
H-atom treatment	H-atom parameters constrained
Δρ_max_, Δρ_min_ (e Å^−3^)	0.51, −0.67

Computer programs: *APEX2* and *SAINT* (Bruker, 2012 ▸), *SHELXT* (Sheldrick, 2015*a* ▸), *SHELXL* (Sheldrick, 2015*b* ▸), *DIAMOND* (Brandenburg, 2006 ▸), *Mercury* (Macrae *et al.*, 2020 ▸), *WinGX* publication routines (Farrugia, 2012 ▸) and *publCIF* (Westrip, 2010 ▸).

## full crystallographic data

### Crystal data

**Table:**

[RuI(C_10_H_14_)(C_10_H_8_N_2_)]PF_6_	*Z* = 2
*M**_r_* = 663.33	*F*(000) = 644
Triclinic, *P*1	*D*_x_ = 1.904 Mg m^−^^3^
Hall symbol: -P 1	Mo *K*α radiation, λ = 0.71073 Å
*a* = 9.3020 (8) Å	Cell parameters from 6634 reflections
*b* = 10.4068 (9) Å	θ = 2.2–20.9°
*c* = 12.0732 (11) Å	µ = 2.14 mm^−^^1^
α = 86.046 (2)°	*T* = 293 K
β = 82.838 (2)°	Block, orange
γ = 88.953 (2)°	0.39 × 0.24 × 0.08 mm
*V* = 1156.80 (18) Å^3^	

### Data collection

**Table:**

Bruker APEX DUO diffractometer	4700 independent reflections
Radiation source: sealed-tube	3595 reflections with *I* > 2σ(*I*)
Triumph monochromator	*R*_int_ = 0.060
Detector resolution: 8.4 pixels mm^-1^	θ_max_ = 26.4°, θ_min_ = 2.0°
φ and ω scans	*h* = −11→11
Absorption correction: multi-scan (SADABS; Krause *et al.*, 2015)	*k* = −12→12
*T*_min_ = 0.657, *T*_max_ = 0.746	*l* = −15→15
41246 measured reflections	

### Refinement

**Table:**

Refinement on *F*^2^	Hydrogen site location: inferred from neighbouring sites
Least-squares matrix: full	H-atom parameters constrained
*R*[*F*^2^ > 2σ(*F*^2^)] = 0.032	*w* = 1/[σ^2^(*F*_o_^2^) + (0.0331*P*)^2^ + 0.7508*P*] where *P* = (*F*_o_^2^ + 2*F*_c_^2^)/3
*wR*(*F*^2^) = 0.077	(Δ/σ)_max_ = 0.003
*S* = 1.05	Δρ_max_ = 0.51 e Å^−^^3^
4700 reflections	Δρ_min_ = −0.67 e Å^−^^3^
345 parameters	Extinction correction: SHELXL (Sheldrick 2015b)
312 restraints	Extinction coefficient: 0.0017 (3)

### Special details

**Table:**

Geometry. All esds (except the esd in the dihedral angle between two l.s. planes) are estimated using the full covariance matrix. The cell esds are taken into account individually in the estimation of esds in distances, angles and torsion angles; correlations between esds in cell parameters are only used when they are defined by crystal symmetry. An approximate (isotropic) treatment of cell esds is used for estimating esds involving l.s. planes.
Refinement. The hydrogen atoms were refined isotropically in their idealized geometrical positions while riding on their anisotropic parent atoms with *U*_iso_ = 1.2*U*_eq_ for the aromatic and methine protons, and *U*_iso_ = 1.5*U*_eq_ for the methyl protons, the latter was refined as a fixed rotor and adjusted to match the hydrogen atoms electron density from the Fourier difference map.The highest electron density of 0.51 e Å^-3^ is 1.17 Å away from F2A, while the deepest electron density of -0.67 e Å^-3^ is 0.76 Å away from I.

### Fractional atomic coordinates and isotropic or equivalent isotropic displacement parameters (Å^2^)

**Table:**

	*x*	*y*	*z*	*U*_iso_*/*U*_eq_	Occ. (<1)
C1	0.5308 (5)	0.5045 (4)	0.7571 (4)	0.0556 (11)	
H1	0.612928	0.518554	0.791235	0.067*	
C2	0.4614 (6)	0.6082 (5)	0.7105 (5)	0.0696 (14)	
H2	0.495887	0.69116	0.712664	0.084*	
C3	0.3415 (6)	0.5875 (5)	0.6610 (5)	0.0762 (16)	
H3	0.293969	0.656742	0.628153	0.091*	
C4	0.2900 (5)	0.4654 (5)	0.6593 (4)	0.0641 (13)	
H4	0.207618	0.450972	0.62566	0.077*	
C5	0.3623 (4)	0.3646 (4)	0.7081 (3)	0.0440 (10)	
C6	0.3159 (4)	0.2297 (4)	0.7163 (3)	0.0426 (9)	
C7	0.1884 (5)	0.1895 (5)	0.6822 (4)	0.0543 (11)	
H7	0.127532	0.248857	0.649623	0.065*	
C8	0.1521 (5)	0.0614 (5)	0.6966 (4)	0.0609 (13)	
H8	0.068448	0.032409	0.671865	0.073*	
C9	0.2417 (5)	−0.0223 (5)	0.7483 (4)	0.0570 (12)	
H9	0.217738	−0.108896	0.76106	0.068*	
C10	0.3674 (5)	0.0218 (4)	0.7812 (4)	0.0507 (11)	
H10	0.427666	−0.036311	0.81581	0.061*	
C11	0.6930 (6)	0.0835 (6)	0.5697 (4)	0.0772 (16)	
H11A	0.667182	−0.005738	0.58185	0.116*	
H11B	0.611672	0.132782	0.547809	0.116*	
H11C	0.773164	0.093052	0.511529	0.116*	
C12	0.7349 (5)	0.1308 (4)	0.6755 (4)	0.0497 (11)	
C13	0.7263 (4)	0.0484 (4)	0.7755 (4)	0.0500 (11)	
H13	0.698556	−0.036743	0.773799	0.06*	
C14	0.7587 (4)	0.0931 (4)	0.8761 (4)	0.0456 (10)	
H14	0.750075	0.037772	0.940359	0.055*	
C15	0.8048 (4)	0.2219 (4)	0.8814 (4)	0.0438 (10)	
C16	0.8137 (4)	0.3028 (4)	0.7834 (4)	0.0478 (10)	
H16	0.843753	0.387401	0.784432	0.057*	
C17	0.7776 (4)	0.2574 (4)	0.6824 (4)	0.0504 (11)	
H17	0.782498	0.313787	0.618903	0.06*	
C18	0.8431 (5)	0.2662 (5)	0.9902 (4)	0.0560 (12)	
H18	0.781336	0.219394	1.050876	0.067*	
C19	0.9994 (6)	0.2249 (7)	1.0001 (5)	0.098 (2)	
H19A	1.026884	0.251901	1.068903	0.147*	
H19B	1.007533	0.132815	0.999577	0.147*	
H19C	1.062047	0.263844	0.938218	0.147*	
C20	0.8194 (6)	0.4082 (5)	1.0047 (5)	0.0799 (16)	
H20A	0.846272	0.42838	1.075707	0.12*	
H20B	0.877795	0.456942	0.945822	0.12*	
H20C	0.719064	0.429642	1.001801	0.12*	
P1A	1.0857 (7)	0.2942 (8)	0.3645 (6)	0.0509 (15)	0.650 (8)
F1A	0.9648 (8)	0.2426 (9)	0.3031 (6)	0.123 (3)	0.650 (8)
F2A	0.9658 (11)	0.3420 (10)	0.4545 (9)	0.131 (4)	0.650 (8)
F3A	1.2007 (9)	0.3421 (12)	0.4310 (6)	0.143 (4)	0.650 (8)
F4A	1.2077 (10)	0.2504 (10)	0.2745 (8)	0.137 (4)	0.650 (8)
F5A	1.0855 (11)	0.1606 (6)	0.4316 (8)	0.159 (4)	0.650 (8)
F6A	1.0734 (12)	0.4250 (7)	0.3012 (9)	0.148 (4)	0.650 (8)
P1B	1.0955 (16)	0.2986 (17)	0.3578 (12)	0.069 (4)	0.350 (8)
F1B	0.992 (2)	0.373 (2)	0.2882 (14)	0.146 (6)	0.350 (8)
F2B	1.1987 (17)	0.3187 (18)	0.2475 (11)	0.101 (5)	0.350 (8)
F3B	1.2132 (17)	0.2278 (16)	0.4181 (13)	0.127 (5)	0.350 (8)
F4B	0.994 (2)	0.283 (2)	0.4678 (17)	0.129 (6)	0.350 (8)
F5B	1.1733 (19)	0.4249 (12)	0.3872 (13)	0.110 (5)	0.350 (8)
F6B	1.060 (2)	0.1721 (12)	0.3120 (14)	0.125 (5)	0.350 (8)
I	0.43771 (3)	0.24106 (3)	1.01101 (2)	0.05001 (11)	
N1	0.4841 (3)	0.3832 (3)	0.7551 (3)	0.0407 (8)	
N2	0.4055 (3)	0.1461 (3)	0.7650 (3)	0.0396 (7)	
Ru	0.59395 (3)	0.22026 (3)	0.80932 (3)	0.03486 (10)	

### Atomic displacement parameters (Å^2^)

**Table:**

	*U*^11^	*U*^22^	*U*^33^	*U*^12^	*U*^13^	*U*^23^
C1	0.052 (3)	0.041 (3)	0.074 (3)	0.000 (2)	−0.013 (2)	−0.001 (2)
C2	0.071 (4)	0.040 (3)	0.097 (4)	0.007 (2)	−0.013 (3)	0.003 (3)
C3	0.079 (4)	0.050 (3)	0.102 (4)	0.013 (3)	−0.029 (3)	0.010 (3)
C4	0.061 (3)	0.059 (3)	0.075 (3)	0.009 (2)	−0.023 (3)	0.000 (3)
C5	0.037 (2)	0.044 (2)	0.052 (2)	0.0039 (18)	−0.0120 (19)	−0.0007 (19)
C6	0.040 (2)	0.045 (2)	0.042 (2)	−0.0011 (19)	−0.0029 (18)	−0.0044 (18)
C7	0.046 (3)	0.068 (3)	0.052 (3)	−0.002 (2)	−0.016 (2)	−0.006 (2)
C8	0.049 (3)	0.072 (3)	0.065 (3)	−0.021 (2)	−0.011 (2)	−0.017 (3)
C9	0.059 (3)	0.057 (3)	0.057 (3)	−0.019 (2)	−0.009 (2)	−0.009 (2)
C10	0.055 (3)	0.045 (3)	0.053 (3)	−0.010 (2)	−0.010 (2)	0.000 (2)
C11	0.090 (4)	0.092 (4)	0.051 (3)	0.016 (3)	−0.006 (3)	−0.026 (3)
C12	0.044 (2)	0.057 (3)	0.046 (2)	0.007 (2)	0.0045 (19)	−0.008 (2)
C13	0.043 (2)	0.038 (2)	0.067 (3)	0.0080 (19)	−0.001 (2)	−0.006 (2)
C14	0.040 (2)	0.045 (2)	0.051 (3)	0.0042 (19)	−0.0067 (19)	0.0052 (19)
C15	0.028 (2)	0.051 (3)	0.052 (2)	−0.0037 (18)	−0.0063 (18)	0.002 (2)
C16	0.031 (2)	0.049 (3)	0.062 (3)	−0.0072 (18)	−0.0017 (19)	0.004 (2)
C17	0.041 (2)	0.059 (3)	0.047 (3)	0.000 (2)	0.0058 (19)	0.006 (2)
C18	0.046 (3)	0.068 (3)	0.056 (3)	−0.010 (2)	−0.015 (2)	−0.004 (2)
C19	0.060 (4)	0.146 (6)	0.097 (5)	0.009 (4)	−0.039 (3)	−0.028 (4)
C20	0.080 (4)	0.079 (4)	0.085 (4)	−0.025 (3)	−0.013 (3)	−0.023 (3)
P1A	0.051 (2)	0.051 (3)	0.050 (3)	−0.004 (2)	−0.006 (2)	−0.004 (2)
F1A	0.100 (5)	0.145 (7)	0.135 (5)	−0.058 (5)	−0.045 (4)	−0.017 (5)
F2A	0.117 (6)	0.130 (8)	0.130 (7)	0.020 (5)	0.055 (5)	−0.023 (6)
F3A	0.135 (6)	0.198 (11)	0.109 (6)	−0.061 (8)	−0.051 (5)	−0.018 (7)
F4A	0.124 (6)	0.134 (8)	0.143 (8)	0.024 (6)	0.043 (5)	−0.050 (6)
F5A	0.189 (8)	0.093 (5)	0.197 (8)	−0.001 (5)	−0.067 (7)	0.059 (5)
F6A	0.178 (9)	0.078 (5)	0.181 (8)	−0.046 (5)	−0.031 (7)	0.069 (5)
P1B	0.075 (6)	0.061 (6)	0.066 (6)	−0.010 (5)	0.014 (5)	−0.010 (5)
F1B	0.115 (11)	0.180 (14)	0.146 (10)	0.071 (10)	−0.043 (9)	−0.008 (11)
F2B	0.099 (9)	0.131 (12)	0.073 (7)	−0.066 (9)	0.008 (6)	−0.010 (7)
F3B	0.122 (10)	0.106 (9)	0.159 (11)	0.026 (9)	−0.056 (8)	0.023 (9)
F4B	0.114 (10)	0.154 (15)	0.095 (8)	0.013 (10)	0.062 (8)	0.032 (10)
F5B	0.150 (11)	0.070 (7)	0.109 (10)	−0.033 (7)	0.008 (9)	−0.030 (7)
F6B	0.148 (11)	0.061 (7)	0.174 (11)	−0.033 (7)	−0.021 (10)	−0.039 (7)
I	0.04595 (18)	0.0559 (2)	0.04652 (18)	−0.00287 (13)	0.00127 (13)	−0.00340 (13)
N1	0.0388 (19)	0.0358 (18)	0.0468 (19)	−0.0039 (14)	−0.0044 (15)	0.0014 (15)
N2	0.0384 (18)	0.0385 (18)	0.0416 (18)	−0.0042 (15)	−0.0047 (14)	−0.0004 (14)
Ru	0.03218 (18)	0.03300 (18)	0.03900 (19)	−0.00260 (13)	−0.00394 (13)	0.00021 (13)

### Geometric parameters (Å, º)

**Table:**

C1—N1	1.346 (5)	C14—H14	0.93
C1—C2	1.371 (6)	C15—C16	1.399 (6)
C1—H1	0.93	C15—C18	1.507 (6)
C2—C3	1.356 (7)	C16—C17	1.417 (6)
C2—H2	0.93	C16—H16	0.93
C3—C4	1.368 (7)	C17—H17	0.93
C3—H3	0.93	C18—C20	1.509 (7)
C4—C5	1.374 (6)	C18—C19	1.525 (7)
C4—H4	0.93	C18—H18	0.98
C5—N1	1.352 (5)	C19—H19A	0.96
C5—C6	1.470 (5)	C19—H19B	0.96
C6—N2	1.350 (5)	C19—H19C	0.96
C6—C7	1.384 (6)	C20—H20A	0.96
C7—C8	1.375 (6)	C20—H20B	0.96
C7—H7	0.93	C20—H20C	0.96
C8—C9	1.368 (7)	P1A—F6A	1.524 (8)
C8—H8	0.93	P1A—F3A	1.525 (8)
C9—C10	1.378 (6)	P1A—F1A	1.546 (8)
C9—H9	0.93	P1A—F4A	1.555 (8)
C10—N2	1.343 (5)	P1A—F2A	1.557 (10)
C10—H10	0.93	P1A—F5A	1.560 (8)
C11—C12	1.499 (6)	P1B—F6B	1.52 (2)
C11—H11A	0.96	P1B—F1B	1.523 (12)
C11—H11B	0.96	P1B—F4B	1.53 (2)
C11—H11C	0.96	P1B—F3B	1.538 (12)
C12—C17	1.394 (6)	P1B—F2B	1.546 (13)
C12—C13	1.426 (6)	P1B—F5B	1.59 (2)
C13—C14	1.398 (6)	I—Ru	2.6958 (5)
C13—H13	0.93	N1—Ru	2.073 (3)
C14—C15	1.423 (6)	N2—Ru	2.072 (3)
			
N1—C1—C2	122.2 (4)	C20—C18—C19	111.9 (4)
N1—C1—H1	118.9	C15—C18—H18	107.4
C2—C1—H1	118.9	C20—C18—H18	107.4
C3—C2—C1	118.8 (5)	C19—C18—H18	107.4
C3—C2—H2	120.6	C18—C19—H19A	109.5
C1—C2—H2	120.6	C18—C19—H19B	109.5
C2—C3—C4	120.4 (5)	H19A—C19—H19B	109.5
C2—C3—H3	119.8	C18—C19—H19C	109.5
C4—C3—H3	119.8	H19A—C19—H19C	109.5
C3—C4—C5	118.8 (5)	H19B—C19—H19C	109.5
C3—C4—H4	120.6	C18—C20—H20A	109.5
C5—C4—H4	120.6	C18—C20—H20B	109.5
N1—C5—C4	121.6 (4)	H20A—C20—H20B	109.5
N1—C5—C6	113.9 (3)	C18—C20—H20C	109.5
C4—C5—C6	124.5 (4)	H20A—C20—H20C	109.5
N2—C6—C7	121.5 (4)	H20B—C20—H20C	109.5
N2—C6—C5	114.4 (3)	F6A—P1A—F3A	92.4 (6)
C7—C6—C5	124.0 (4)	F6A—P1A—F1A	89.7 (5)
C8—C7—C6	119.7 (4)	F3A—P1A—F1A	176.9 (6)
C8—C7—H7	120.2	F6A—P1A—F4A	91.5 (7)
C6—C7—H7	120.2	F3A—P1A—F4A	89.5 (5)
C9—C8—C7	118.5 (4)	F1A—P1A—F4A	92.8 (6)
C9—C8—H8	120.7	F6A—P1A—F2A	87.3 (7)
C7—C8—H8	120.7	F3A—P1A—F2A	89.4 (6)
C8—C9—C10	119.9 (4)	F1A—P1A—F2A	88.4 (6)
C8—C9—H9	120	F4A—P1A—F2A	178.3 (8)
C10—C9—H9	120	F6A—P1A—F5A	175.6 (7)
N2—C10—C9	122.0 (4)	F3A—P1A—F5A	90.3 (5)
N2—C10—H10	119	F1A—P1A—F5A	87.5 (6)
C9—C10—H10	119	F4A—P1A—F5A	92.0 (7)
C12—C11—H11A	109.5	F2A—P1A—F5A	89.3 (6)
C12—C11—H11B	109.5	F6B—P1B—F1B	91.8 (13)
H11A—C11—H11B	109.5	F6B—P1B—F4B	97.1 (14)
C12—C11—H11C	109.5	F1B—P1B—F4B	98.2 (13)
H11A—C11—H11C	109.5	F6B—P1B—F3B	89.1 (13)
H11B—C11—H11C	109.5	F1B—P1B—F3B	173.7 (16)
C17—C12—C13	117.1 (4)	F4B—P1B—F3B	87.9 (12)
C17—C12—C11	122.1 (4)	F6B—P1B—F2B	84.6 (11)
C13—C12—C11	120.8 (4)	F1B—P1B—F2B	81.6 (12)
C14—C13—C12	121.3 (4)	F4B—P1B—F2B	178.3 (18)
C14—C13—H13	119.3	F3B—P1B—F2B	92.3 (11)
C12—C13—H13	119.3	F6B—P1B—F5B	164.9 (14)
C13—C14—C15	121.0 (4)	F1B—P1B—F5B	93.7 (14)
C13—C14—H14	119.5	F4B—P1B—F5B	96.1 (14)
C15—C14—H14	119.5	F3B—P1B—F5B	83.9 (11)
C16—C15—C14	117.8 (4)	F2B—P1B—F5B	82.3 (11)
C16—C15—C18	122.4 (4)	C1—N1—C5	118.2 (4)
C14—C15—C18	119.8 (4)	C1—N1—Ru	124.5 (3)
C15—C16—C17	120.8 (4)	C5—N1—Ru	117.1 (3)
C15—C16—H16	119.6	C10—N2—C6	118.4 (4)
C17—C16—H16	119.6	C10—N2—Ru	124.6 (3)
C12—C17—C16	122.0 (4)	C6—N2—Ru	117.1 (3)
C12—C17—H17	119	N2—Ru—N1	76.86 (12)
C16—C17—H17	119	N2—Ru—I	84.69 (9)
C15—C18—C20	114.7 (4)	N1—Ru—I	86.99 (9)
C15—C18—C19	107.6 (4)		
			
N1—C1—C2—C3	0.2 (8)	C18—C15—C16—C17	179.6 (4)
C1—C2—C3—C4	0.9 (9)	C13—C12—C17—C16	1.1 (6)
C2—C3—C4—C5	−0.2 (8)	C11—C12—C17—C16	178.4 (4)
C3—C4—C5—N1	−1.5 (7)	C15—C16—C17—C12	−1.4 (6)
C3—C4—C5—C6	177.4 (5)	C16—C15—C18—C20	29.0 (6)
N1—C5—C6—N2	−2.7 (5)	C14—C15—C18—C20	−151.5 (4)
C4—C5—C6—N2	178.3 (4)	C16—C15—C18—C19	−96.3 (5)
N1—C5—C6—C7	174.6 (4)	C14—C15—C18—C19	83.2 (5)
C4—C5—C6—C7	−4.4 (7)	C2—C1—N1—C5	−1.9 (7)
N2—C6—C7—C8	−0.8 (6)	C2—C1—N1—Ru	173.6 (4)
C5—C6—C7—C8	−177.9 (4)	C4—C5—N1—C1	2.5 (6)
C6—C7—C8—C9	2.2 (7)	C6—C5—N1—C1	−176.5 (4)
C7—C8—C9—C10	−2.0 (7)	C4—C5—N1—Ru	−173.2 (3)
C8—C9—C10—N2	0.4 (7)	C6—C5—N1—Ru	7.7 (4)
C17—C12—C13—C14	0.3 (6)	C9—C10—N2—C6	1.0 (6)
C11—C12—C13—C14	−177.0 (4)	C9—C10—N2—Ru	−178.9 (3)
C12—C13—C14—C15	−1.5 (6)	C7—C6—N2—C10	−0.8 (6)
C13—C14—C15—C16	1.2 (6)	C5—C6—N2—C10	176.5 (4)
C13—C14—C15—C18	−178.2 (4)	C7—C6—N2—Ru	179.1 (3)
C14—C15—C16—C17	0.2 (6)	C5—C6—N2—Ru	−3.5 (4)

### Hydrogen-bond geometry (Å, º)

**Table:**

*D*—H···*A*	*D*—H	H···*A*	*D*···*A*	*D*—H···*A*
C1—H1···F2*B*^i^	0.93	2.44	3.136 (13)	132
C7—H7···F4*B*^ii^	0.93	2.65	3.41 (2)	140
C9—H9···F1*A*^iii^	0.93	2.45	3.159 (7)	134
C10—H10···I^iv^	0.93	3.23	4.131 (5)	164

Symmetry codes: (i) −*x*+2, −*y*+1, −*z*+1; (ii) *x*−1, *y*, *z*; (iii) −*x*+1, −*y*, −*z*+1; (iv) −*x*+1, −*y*, −*z*+2.
